# A review of 14 cases of perianal Paget’s disease: characteristics of anorectal cancer with pagetoid spread

**DOI:** 10.1186/s12957-022-02872-z

**Published:** 2023-01-20

**Authors:** Jun Imaizumi, Konosuke Moritani, Yasuyuki Takamizawa, Manabu Inoue, Shunsuke Tsukamoto, Yukihide Kanemitsu

**Affiliations:** Department of Colorectal Surgery, National Cancer Center Hospital, 5-1-1 Tsukiji, Chuo-ku, Tokyo, 104-0045 Japan

**Keywords:** Pagetoid spread, Extramammary paget’s disease, Anorectal cancer, CK20, GCDFP-15, CDX-2

## Abstract

**Background:**

Perianal Paget’s disease (PPD) is an intraepithelial invasion of the perianal skin and is frequently associated with underlying anorectal carcinoma. The relatively rare nature of this disease has made it difficult to develop treatment recommendations. This study aims to analyze the clinical and pathological features of perianal Paget’s disease (PPD) and to explore rational treatment options and follow-up for this disease.

**Methods:**

The National Cancer Center Hospital database was searched for all cases of perianal Paget’s disease diagnosed between 2006 and 2021. In the 14 patients identified, we reviewed the diagnosis, management, and outcomes of adenocarcinoma with pagetoid spread, including suspected or recurrent cases.

**Results:**

All 14 cases met the inclusion criteria. The median follow-up period after diagnosis was 4.5 (range, 0.1–13.0) years. Pagetoid spread before initial treatment was suspected in 12 cases (85.7%). Underlying rectal cancer was identified in 6 cases, and no primary tumor was detected in the other 6 cases. Seven patients had recurrent disease, with the median time to recurrence of 34.6 (range, 19.2–81.7) months. The time to the first relapse was 3 months, and that to the second relapse was 6 months. The overall 5-year survival rate was 90.0%.

**Conclusions:**

Endoscopic and radiologic evaluation, as well as immunohistologic examination, should be performed. is to differentiate PPD with and without underlying anorectal carcinoma. The time to first recurrence varies widely, and long-term and regular follow-up for more than 5 years is considered necessary for local recurrence and distant metastasis.

## Background

Pagetoid spread (PS) is defined as the proliferation of individual cells in the epithelium characterized by erythema and inflammation. It can manifest as mammary or extramammary Paget’s disease (EMPD) and is described as an apocrine gland tumor that can be benign or malignant with metastatic potential. EMPD has been reported at several extramammary sites, including the axilla, thigh, groin, perineum, scrotum, vulva, and perianal area [1, 2]. When EMPD affects the perianal region, it is called perianal Paget’s disease (PPD). PPD is usually associated with an underlying malignancy, such as anal, rectal, cervical, or urinary bladder adenocarcinoma, with an appearance similar to that of simple PPD without an underlying malignancy [3, 4]. The prognosis of simple PPD is relatively favorable, with overall and disease-free survival of approximately 60% at 5 years [5–7]. However, PPD associated with an underlying malignancy has been reported to have a poor prognosis [5–9].

Immunohistochemical analysis of the skin lesion is useful for differentiating whether or not PPD is associated with underlying anorectal adenocarcinoma or carcinoma. Immunohistochemical markers that are frequently used include cytokeratin 7 (CK7), CK20, gross cystic disease fluid protein 15 (GCDFP-15), and CDX2. Although CK7 is a sensitive marker for almost all pagetoid neoplasms, it is not practical for differentiating PPD because some rectal adenocarcinomas also express CK7 [10, 11]. CK20 might be expressed in colorectal carcinoma but not in simple PPD [12]. GCDFP-15, which is regarded as a specific marker for apocrine epithelium tissue, is usually positive in simple PPD [13–15]. As mentioned above, PPD associated with anorectal carcinoma often has the CK20+/GCDFP-15− immunophenotype. In contrast, simple PPD shows a CK20−/GCDFP-15+ pattern [16]. CDX2, which has a high positivity rate in gastrointestinal cancers, including rectal cancer, has been used [17]. However, about 17% of colorectal cancers are negative for CK20 [18]. Therefore, it is necessary to evaluate the underlying malignancy using a combination of colonoscopy and radiology [19].

### Staging classification and treatment outcome

A staging classification for PPD that includes appropriate treatment for each stage has been proposed (Table 1) [9, 20]. The prognosis is good for stage I (Paget’s cells found in the perianal epidermis and adnexa without primary carcinoma) but worsens for stage II (invasive cutaneous disease penetrating the basement membrane and entering the underlying stroma and/or synchronous localized malignancies, i.e., IIa adnexal malignancy and IIb visceral malignancy), stage III (regional metastatic disease), and stage IV (distant metastatic disease).

**Table 1:** Perianal Paget’s disease classification and accompanying suggested therapy

Stage	Description	Management
I	Paget’s cells in the perianal epidermis and adnexa without primary carcinoma	Wide local excision
IIA	Cutaneous Paget’s disease with associated adnexal carcinoma	Wide local excision
IIB	Cutaneous Paget’s disease with associated anorectal carcinoma	Abdominoperineal resection
III	Paget’s disease in which associated carcinoma has spread to regional nodes	Inguinal lymph nodes dissection and abdominoperineal resection/wide local excision
IV	Paget’s disease with distant metastases of associated carcinoma	Chemotherapy, radiotherapy, local palliative management

*APR* abdominal perineal rectal dissection, *WLE* wide local excision

The treatment options for PPD depend on local (extent and depth of invasion) and regional (lymph node involvement) factors and the extent of systemic disease. Local excision (with macroscopic clearance of surgical margins) has been performed as a treatment for non-invasive PPD but was associated with a high local recurrence rate (40%) [19]. Wide local excision (WLE, >1 cm microscopic clearance of surgical margins) with a sphincter-saving technique was later proposed as the treatment of choice for PPD due to the higher survival rate in patients treated with WLE than in those treated with local excision and due to the better chance of cure and normal survival. The standard treatment for PPD associated with the anorectal canal is abdominoperineal resection (APR) [9, 20]. However, it is recommended that adenocarcinoma of the anal canal be managed in the same way as rectal cancer [21], and transanal local excision is appropriate for selected early-stage rectal cancers [22]. Therefore, even in cases of PPD associated with anal canal cancer, combined transanal local excision and WLE may be an alternative to APR.

The distinction between PPD with and without underlying anorectal cancer is essential [3, 23–25] because of the differences in treatment methods and the prognosis [4]. Due to the rarity of PPD, few cases have been reported [24–27], and long-term outcomes of treatment for PS are unknown. This study aims to analyze the clinical and pathological features of perianal Paget’s disease (PPD) and to explore rational treatment options and follow-up for this disease.

## Methods

A search of the National Cancer Center Hospital database identified 14 cases of PPD diagnosed between 2006 and 2021. Cases of adenocarcinoma of the rectum or anal canal with pagetoid spread, including suspected and recurrent cases, were retrospectively reviewed for age, sex, tumor size, presence or absence of preoperative endoscopic findings, presence or absence of mapping biopsy, treatment methods, and long-term prognosis.

The study was approved by the National Cancer Center Hospital Institutional Review Board (code: 2017–437). The requirement for written informed consent was waived in view of the retrospective nature of the research and the anonymity of the study data.

## Results

All 14 patients met the inclusion criteria. Eight patients (57.1%) were male, and 7 (50.0%) were female (Table 2). The median age was 74 (range, 55–84) years. Twelve patients (85.7%) were suspected of having PS before initial treatment. The most commonly used immunostaining for differentiation was CK7+/CK20+/GCDFP-15−/CDX2+. These 12 patients were clinically and radiologically evaluated for underlying anorectal carcinoma, which was identified in 6 cases. The median size of the primary tumor was 52 (9–110) mm. No primary lesions were detected in the other 6 cases. There were no distant metastases at the initial diagnosis; however, 1 patient had metastasis to the lateral lymph nodes, and another had metastasis to the inguinal lymph nodes.

**Table 2:** Clinicopathologic characteristics

Characteristic	Value	(*n*=14)
Age, median (IQR), years	74	(67.5–78.5)
Gender		
Male, No. (%)	7	(50%)
Female, No. (%)	7	(50%)
Preoperative diagnosis of pagetoid spread, No. (%)		
Diagnosed	2	(14%)
Not diagnosed	12	(86%)
Identification of underlying anorectal cancer, No. (%)		
Not found	6	(43%)
Syncronous	7	(50%)
Metachronous	1	(7%)
Primary tumor site, No. (%)		
Anal canal	7	(50%)
Lower rectum	1	(7%)
Unknown	6	(43%)
Immunohistochemistry, No. (%)		
Not confirmed	2	(14%)
CK20+/CK7+	7	(50%)
CK20+/CK7−	4	(29%)
CK20−/CK7+	0	(0%)
CK20−/CK7−	1	(7%)
GCDFP15−^a^	8	(100%)
CDX2+	11	(92%)
Histology^a^, No. (%)		
Adenocarcinoma (grade unknown)	7	(50%)
Tub1/2	1	(7%)
Por/sig	4	(933%)
Muc	3	(600%)
Tumor size, median (IQR), cm	53.5	(26.3–87.5)
Depth of invasion, No. (%)		
M	3	(21%)
SM	1	(7%)
MP	2	(14%)
A	1	(7%)
AI	1	(7%)
Invasive (WLE)	1	(7%)
Non-invasive (WLE)	2	(14%)
Lymph node metastases at initial treatment, No. (%)		
Negative	4	(29%)
Lateral lymph node	1	(7%)
Inguinal lymph node	2	(14%)
Unknown (clinically negative)	7	(50%)
Initial treatment, No. (%)		
WLE (±transanal local excision	7	(50%)
APR (±WLE)	5	(36%)
Radiation	1	(7%)
Unknown^b^	1	(7%)
Mapping biopsy^c^, No. (%)		
Done	5	(42%)
None	7	(58%)
Resection status^c^, No. (%)		
R0		(0%)
R1		(0%)
R2		(0%)
Follow-up time, median (IQR), mo	4.5	(158.7–7.4)
Recurrence, No (%)	7	(50%)
Time to recurrence, median (IQR), mo	34.6	(18.2–81.7)
5-year recurrence free survival	55.9%	
Mortality, No. (%)	2	(14%)
5-year overall survival	90.0%	

Abbreviation: *IQR* interquartile range, *WLE* wide local excision, *RT* radiotherapy, *LE* local excision, *APR* abdominoperineal resection

^a^There are duplicates

^b^Referred to another hospital

^c^Excluded: non-surgical 2 cases

The median follow-up period after diagnosis was 4.5 (range, 0.1–13.0) years. At the last follow-up, 7 patients were alive with no evidence of disease, 4 were alive with recurrent disease, 1 had transferred to another hospital, and 2 had died of disease. A summary of the 13 cases and their clinical course is presented in Table 3.

**Table 3:** A summary of cases attended

Case	Gender	Initial treatment	Immunohistological findings				Primary	Histology*	LN mets	Mapping biopsy	1st relapse		Outcome
	Age	Lymph node dissection	CK7	CK20	GCDFP15	CDX2	Maximum diameter	Depth of invasion	Location of LN	Resection status	Interval	Treatment	Follow-up time (primary)
No detectable primary lesion													
1	M	WLE	−	+	NA	+	Unknown	Adenocarcinoma	Negative	Done	ILN	ILND	Dead of disease
	76	None					55mm	Invasive		R1	61 months		6 years
2	M	WLE	+	+	−	+	Unknown	Adenocarcinoma	NA	Done	ILN	ILND	Alive with no evidence of disease
	55	None					30mm	Non-invasive		R1	34 months		13 years
3	F	RT	−	+	−	−	Unknown	Adenocarcinoma	NA	None	Local	Medication	Alive with 2nd relapse
	78	None						NA		NA	87 months		12 years
4	F	APR with WLE	+	+	−	+	Unknown	Adenocarcinoma	Negative	None	NA	NA	Alive with no evidence of disease
	73	D2					74mm	MP		R0			5 years
5	F	WLE	−	−	NA	−	Unknown	Muc	Positive	Done	NA	NA	Alive with no evidence of disease
	63	ILND					80mm	Invasive	Inguinal	R0			2 years
6	M	WLE	+	+	−	−	Unknown	Adenocarcinoma	Negative	None	Local	WLE	Alive with 2nd relapse
	80	None					50mm	Invasive		R0	25 months		5 years
Detectable underlying anorectal carcinoma													
7	M	APR with WLE	−	+	NA	+	*P*	Muc/sig	Positive	None	ILN	ILND	Dead of disease
	68	D3,LLND					110mm	AI	Lateral pelvic	R1	7 months		2 years
8	M	Transanal LE with WLE	−	+	−	+	*P*	Tub1	NA	None	NA		Alive with no evidence of disease
	72	None					9mm	SM1		R0			5 years
9	F	APR with WLE	+	+	−	+	*P*	Tub1/por/sig	Negative	Done	LD/ILN	LLND	Alive with no evidence of disease
	80	D2					90mm	MP		R1	13 months	ILND	2 years
10	M	APR with WLE	+	+	NA	+	*P*	Adenocarcinoma	Negative	None	NA		Alive with no evidence of disease
	66	D2					106mm	M		R0			3 years
11	F	NA	+	+	−	+	Rb	NA	NA	NA	NA		Unknown
	69	None					Unknown						3 years
12	F	Transanal LE with WLE	+	+	−	+	*P*	Por2/tub2/muc	NA	Done	Local	WLE	Alive with no evidence of disease
	84	None					52mm	M		R1	36 months		4 years
No preoperative diagnosis of pagetoid spread													
13	M	Trans anal LE*	NA	NA	NA	NA	*P*	Tub2/por	NA	NA	Local	WLE	Alive with 2nd relapse
	74	None					10mm	SM			159 months		13 years
14	F	Chemoterapy	NA	NA	NA	+	*P*	Por/sig	Positive	None	NA		Alive with
	74	D3, LLND, ILND					25mm	A	Inguinal	Positive			1 year

*APR* abdominal perineal rectal dissection, *WLE* wide local excision, *LLND* lateral lymph node dissection, *ILND* inguinal lymph node dissection

Seven patients (50%) developed recurrence, with a median time to recurrence of 34.6 (range 19.2–81.7) months. Three patients had local recurrence, and 4 (28.6%) had a recurrence in the inguinal lymph nodes. One patient had distant metastases. The time to the first relapse was 3 months, and that to the second relapse was 6 months. The 5-year overall survival rate was 90.0%.

The initial treatment in the 6 patients with no detectable primary lesion was WLE alone (*n*=4, 66.7%), APR + WLE (*n*=1, 16.7%), and radiotherapy (*n*=1, 16.7%). The outcomes in the 4 patients who underwent WLE alone were as follows: alive with no evidence of disease (*n*=2), alive with lung metastasis (*n*=1), and died after inguinal lymph node metastases at first relapse and liver and bone metastases at the second relapse (*n*=1).

The initial treatment of the 6 patients in whom anorectal carcinoma was detected was APR + WLE (*n*=3, 50%), transanal local excision + WLE (*n*=2, 33.3%), and referral to another hospital (*n*=1, 16.7%). The outcomes in the group that underwent APR + WLE were as follows: alive with no evidence of disease (*n*=2) and dead (*n*=1). The outcomes in the group that underwent transanal local excision + WLE were as follows: alive with no evidence of disease (*n*=1) and alive with recurrent disease (*n*=1).

In terms of initial treatment, there were 4 recurrences after WLE (with or without transanal local excision), 2 after APR, and 1 after radiotherapy. All 6 cases of recurrence after surgical resection had positive resection margins at the initial surgery. Mapping biopsy was performed in 5 (45.4%) of the 11 patients who underwent surgical resection, and 4 (80.0%) had positive margins. In the 6 cases without mapping biopsy, 2 (33.3%) had positive margins.

## Discussion

PPD is usually associated with an underlying malignant anorectal tumor and has a relatively poor prognosis [23] with a high risk of local recurrence [28]. The rate of malignancy associated with PPD ranges from 33 to 86% [29]. In the present study, of the 11 cases of PPD associated with anal canal cancer who presented with CK20+/GCDFP-15− (GCDFP was not examined in some cases), anal cancer could be noted in 6 patients by endoscopic or radiographic evaluation. Immunohistological examination alone is not sufficient to identify underlying malignancy, and it is critical to apply PD staging (Table 1) in conjunction with endoscopic and radiologic evaluation.

In the present study, primary anorectal carcinoma could not be identified preoperatively in half of the patients, and APR was performed in only 1 case. There was 1 death in the WLE group. However, only half of the patients with anorectal cancer underwent APR + WLE to preserve the anus, although there were cases of recurrence-free survival of more than 5 years after WLE. Further investigations are needed to identify cases in which APR should be pursued aggressively and those in which WLE (with or without transanal local excision) can be considered.

The 2 deaths occurred in a patient without an identified primary tumor who underwent WLE alone as the initial treatment (case 1) and in a patient with a primary tumor identified preoperatively who underwent APR + WLE (case 7).

Of the 6 cases in which anal cancer was detected, APR + WLE was performed in 3 patients and Transanal Local Excision was performed in 2 cases. In the latter two cases, the depth of the primary tumor was M/SM1, and recurrence-free survival of 2 to 5 years was achieved. Further investigation is considered to be necessary.

On the other hand, both these fatal cases had inguinal lymph node metastasis as the first relapse, and distant metastasis several months later.

The time to first recurrence varies from 7 months to about 5 years, and it is difficult to predict prognosis in general from the course of the disease, but long-term and regular follow-up for more than 5 years is considered necessary to check for recurrence and distant metastasis.

Intraoperative frozen section analysis of the resection margin has been proposed to reduce the possibility of borderline invasion and minimize the local recurrence rate [19]. However, frozen section analysis of surgical margins in PPD can be misleading and dangerous because it may appear negative intraoperatively but become permanently positive on subsequent histological analysis. It is believed that permanent margin status is not a predictor of local recurrence and that a minimally invasive carcinoma measuring <1 mm probably does not have an adverse prognosis, whereas a deeply invasive carcinoma behaves as a fully malignant adenocarcinoma [30]. Of the five patients who underwent mapping biopsy in this study, four were positive for transection, but only one case resulted in local recurrence. This is consistent with previous studies that have shown that edge evaluation is not a predictor of local recurrence.

There are several potential limitations to this study. First, there is the possibility of selection bias due to the retrospective study design. Second, the sample size is very small (14 cases) and cannot shed light on treatment possibilities, especially the optimal approach and prognosis. Despite these limitations, we believe that our findings warrant more extensive investigation in patients with PPD.

## Conclusions

Although skin biopsy and immunohistological diagnosis are useful for distinguishing underlying malignancy in patients with PPD, endoscopic and radiologic evaluation is mandatory. The time to first recurrence varies widely, and long-term and regular follow-up for more than 5 years is considered necessary for local recurrence and distant metastasis.

## Data Availability

All data generated or analyzed during this study are included in this published article.

## Abbreviations

CI: Confidence interval
EMPD: Extramammary Paget’s disease
HR: Hazard ratio
PPD: Perianal Paget’s disease
PS: Pagetoid spread
